# Editorial for the Special Issue “Gels for Energy Applications”

**DOI:** 10.3390/gels12100938

**Published:** 2026-10-20

**Authors:** Panpan Zhao, Miguel Sanchez-Soto

**Affiliations:** 1 Department of Molecular Chemistry and Materials Science, Weizmann Institute of Science, Rehovot 76100, Israel; 2 e-Plascom Research Group, Department of Materials Science and Engineering, Universitat Politècnica de Catalunya-Barcelona Tech (UPC), Escola d’Enginyeria de Barcelona Est (EEBE), Campus Diagonal-Besòs, Av. D’Eduard Maristany, 16, 08019 Barcelona, Spain; 3 Centre for Research in Multiscale Science and Engineering of Barcelona, Universitat Politècnica de Catalunya-Barcelona Tech (UPC), Escola d’Enginyeria de Barcelona Est (EEBE), Campus Diagonal-Besòs, Av. D’Eduard Maristany, 16, 08019 Barcelona, Spain

Gels have emerged as versatile materials for energy-related applications because their 3D networks can act as supporting materials with structural stability, flexibility, and tunable thermal and interfacial properties. These characteristics make them attractive for electrochemical energy storage, thermal management, energy conversion, and safety-related technologies.

This Special Issue, “Gels for Energy Applications”, highlights recent advances demonstrating the broad potential of gel-based materials in energy science and engineering. Sutradhar et al. reviewed the development of hydrogels for next-generation rechargeable batteries, emphasizing their advantages in ionic transport, flexibility, and multifunctionality across different battery chemistries (contribution 1). Viggiano et al. developed highly porous polyimide gels for use as battery separators with room-temperature ionic-liquid electrolytes, providing a promising approach to improving thermal stability and battery safety (contribution 2).

Thermal energy management is represented by the work of Zhao et al., who reviewed aerogel-supported phase change materials with particular emphasis on flame-retardant strategies (contribution 3). Their contribution highlights the ability of porous gel-derived frameworks to integrate thermal energy storage, structural stabilization, and fire-safety functions. In the field of energy conversion, Bhuyan and Jeong reviewed functional hydrogel-based flexible thermoelectric generators, discussing their working mechanisms, material design, and potential applications in low-grade heat harvesting and wearable devices (contribution 4).

The role of gels in energy-related safety is further demonstrated by Wang et al., who developed a carboxymethyl-cellulose-based composite gel with both flame-retardant and dust-suppression functions for coal-related applications (contribution 5). This work broadens the scope of gel materials beyond energy storage and conversion toward safer energy utilization.

Overall, the contributions to this Special Issue demonstrate the versatility of gels as multifunctional materials capable of integrating transport, mechanical adaptability, thermal regulation, and safety functions. Continued advances in network design, interfacial control, stability, and scalable fabrication are expected to further expand the role of gel-based materials in sustainable and safe energy technologies.

